# Pocket Brain Review

**DOI:** 10.3389/fneur.2012.00143

**Published:** 2012-10-30

**Authors:** Lawrence Korngut

**Affiliations:** ^1^Department of Clinical Neurosciences, Hotchkiss Brain Institute, University of CalgaryCalgary, AB, Canada

Neuroanatomy teaching most often takes the form of detailed didactic lectures, consolidated by application of new knowledge in the anatomy laboratory where cadaveric specimens can be closely examined, and are often accompanied by clinical vignettes and imaging correlates. Such exposure to neuroanatomy is undoubtedly effective, but for learners then proceeding to further training where neuroanatomy is frequently relevant, portable, accessible, and accurate learning aids are invaluable. The emergence and now ubiquitous availability of smartphones and tablets provides an opportunity for all areas of medical education, and may be particularly amenable to the areas requiring memorization of structures in three dimensions, such as anatomy.

Pocket Brain is a new application from eMedia Interactive Ltd. that aims to facilitate neuroanatomy education, through the use of interactive 3D multi-layer models and labeled cross sections of the brain. The app also includes labeled cross sections and animations of the four major tracts. Maneuvering through the application is intuitive, and as tested (version 1.2 on the iPhone 4s with iOS 6.0, and on the iPad 1 with iOS 5.1.1) the application runs well without hang time or crashes. Where the iPhone version shines, the iPad version is even better for demonstration and discussion purposes with its larger screen.

**Figure 1:**
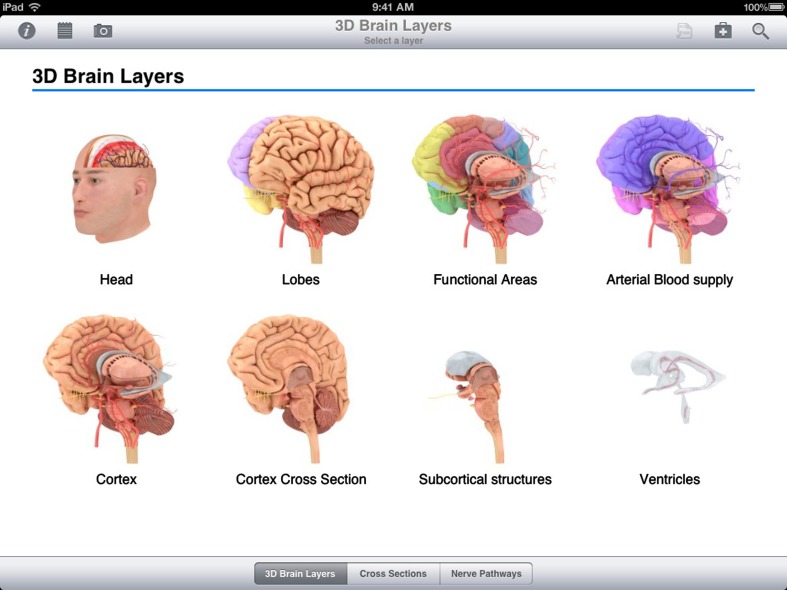
**Snapshot of 3D Brain Layers within the Pocket Brain application**.

Pocket Brain includes a glossary that is aimed at a relatively introductory level (i.e., anatomy students, medical students, nursing, and allied health) as well as eight clinical cases that allow the user to localize the lesion and form a diagnosis. The ability to view the brain from different angles in three-dimensional space and to peel off layers in the process makes it useful for illustrating the relative locations of structures (i.e., arterial supply and cranial nerves when discussing aneurysms). However, the level of detail available is limited, particularly on the labeling of vascular structures, which limits the app’s usefulness as a learning tool beyond the level of senior medical or anatomy student, and restricts its utility for localization at the clinic or ward. Similarly, the cross-sectional views are simplified, with a sufficient level of detail for introductory learners, but not for those beyond the senior medical or anatomy student level.

I would recommend Pocket Brain for students learning neuroanatomy at the introductory level. For residents and consultants in neurology and neurosurgery, though, Pocket Brain will have limited utility in the clinic and at the bedside from a teaching perspective. The true potential of this application is in future version updates. The foundation for a broadly useful application for both medical education as well as clinical localization is there. Future updates could include more detailed vascular anatomy (i.e., middle cerebral artery branch labels with vascular territories for vascular lesion localization), connectivity between nuclei (i.e., movement disorder pathways), and cerebrospinal fluid flow patterns (including common points of obstruction and the clinical consequences). Anatomical details for specific clinical syndromes (i.e., internuclear ophthalmoplegia) would make Pocket Brain more useful to senior learners.

Pocket Brain is among the first useful applications for neuroanatomy education, but the limitations cited above should be considered. While certainly useful for students new to neuroanatomy, the potential for more anatomical detail in future updates makes this application worth watching for expanded appeal to neurologists and to the broader neurosciences community.

